# Wheat Soil-Borne Mosaic Virus Disease Detection: A Perspective of Agricultural Decision-Making via Spectral Clustering and Multi-Indicator Feedback

**DOI:** 10.3390/plants14152260

**Published:** 2025-07-22

**Authors:** Xue Hou, Chao Zhang, Yunsheng Song, Turki Alghamdi, Majed Aborokbah, Hui Zhang, Haoyue La, Yizhen Wang

**Affiliations:** 1 School of Computer and Information Technology, Shanxi University, Taiyuan 030006, China; houxue@sxu.edu.cn (X.H.); wangyizhen@sxu.edu.cn (Y.W.); 2 College of Information Science and Engineering, Shandong Agricultural University, Taian 271018, China; songys@sdau.edu.cn; 3 Faculty of Computer, Islamic University of Madinah, Madinah 42351, Saudi Arabia; dr.turki2@iu.edu.sa; 4 Faculty of Computers and Information Technology, University of Tabuk, Tabuk 71491, Saudi Arabia; m.aborokbah@ut.edu.sa; 5 iGALAXY Information Co., Ltd., Taiyuan 030006, China; zhanghui@igalaxycn.com (H.Z.); lahy@igalaxycn.com (H.L.)

**Keywords:** agricultural artificial intelligence, plants disease assessment, social network

## Abstract

The rapid advancement of artificial intelligence is transforming agriculture by enabling data-driven plant disease monitoring and decision support. Soil-borne mosaic wheat virus (SBWMV) is a soil-transmitted virus disease that poses a serious threat to wheat production across multiple ecological zones. Due to the regional variability in environmental conditions and symptom expressions, accurately evaluating the severity of wheat soil-borne mosaic (WSBM) infections remains a persistent challenge. To address this, the problem is formulated as large-scale group decision-making process (LSGDM), where each planting plot is treated as an independent virtual decision maker, providing its own severity assessments. This modeling approach reflects the spatial heterogeneity of the disease and enables a structured mechanism to reconcile divergent evaluations. First, for each site, field observation of infection symptoms are recorded and represented using intuitionistic fuzzy numbers (IFNs) to capture uncertainty in detection. Second, a Bayesian graph convolutional networks model (Bayesian-GCN) is used to construct a spatial trust propagation mechanism, inferring missing trust values and preserving regional dependencies. Third, an enhanced spectral clustering method is employed to group plots with similar symptoms and assessment behaviors. Fourth, a feedback mechanism is introduced to iteratively adjust plot-level evaluations based on a set of defined agricultural decision indicators sets using a multi-granulation rough set (ADISs-MGRS). Once consensus is reached, final rankings of candidate plots are generated from indicators, providing an interpretable and evidence-based foundation for targeted prevention strategies. By using the WSBM dataset collected in 2017–2018 from Walla Walla Valley, Oregon/Washington State border, the United States of America, and performing data augmentation for validation, along with comparative experiments and sensitivity analysis, this study demonstrates that the AI-driven LSGDM model integrating enhanced spectral clustering and ADISs-MGRS feedback mechanisms outperforms traditional models in terms of consensus efficiency and decision robustness. This provides valuable support for multi-party decision making in complex agricultural contexts.

## 1. Introduction

The increasing complexity of modern agriculture, driven by climate variability, pathogen selection, and shifting agro-ecological systems, has challenged traditional approaches to crop disease monitoring and control [1,2]. Conventional methods that rely on manual observation and expert judgment often suffer from subjectivity, inconsistency, and limited scalability, especially in large-scale production systems [3]. In this context, the rapid advancement of artificial intelligence (AI) technologies are reshaping agriculture, bringing unprecedented capabilities in monitoring, automated control, and decision making [4,5,6]. By harnessing powerful algorithms for data processing, pattern recognition, and predictive analytics, AI enables more precise detection of plant diseases, forecasting of yield trends, and optimization of resource usage [7]. Through the integration of plant health indicators, environmental conditions, soil characteristics, and historical disease data, AI can deliver timely and actionable insights for farmers, agronomists, and policymakers, laying the foundation for sustainable food production systems [8].

According to the latest revision of the United Nations World Population (https://www.un.org/, accessed on 6 April 2025) Prospects, the global population is projected to grow substantially from 7.8 billion in 2020 to approximately 11 billion by 2100. The Food and Agriculture Organization (FAO) (https://www.fao.org/home/en/, accessed on 6 April 2025) forecasts additionally that this figure will reach 9.6 billion by 2050. As a result, global food systems must undergo substantial production increases to accommodate the exponentially rising needs of this expanding population. Approximately 40% of crops worldwide are lost before reaching the market each year, resulting in an estimated loss of USD 540 billion for farmers [9] and among these, plant diseases are responsible for an estimated 15% to 25% of global agricultural production losses each year [10]. Among them, soil-borne wheat mosaic virus (SBWMV) ranks as one of the most persistent and economically devastating pathogens affecting cereal crops worldwide. As such, early detection and effective management of plant infectious diseases are essential to ensuring food security and minimizing economic losses.

Wheat is a primary source of calories and protein worldwide, serving as a staple food for approximately one-third of the global population. As one of the most important food crops, wheat is cultivated annually over 219 million hectares, producing more than 760 million tonnes [11]. However, wheat production is challenged by a range of mechanical, physiological, and biological stresses that affect plants throughout their life cycle and in various environmental conditions. These stresses make wheat crops vulnerable to numerous diseases, which significantly reduce overall yield [12,13]. Among these, over 100 distinct diseases caused by different pathogens and pests impact wheat, leading to an estimated annual loss of approximately 21.5% of the total wheat yield [14], among which the wheat soil-borne mosaic (WSBM) poses a growing concern.

WSBM is a destructive viral disease affecting wheat production across temperate regions worldwide, particularly in areas with autumn-sown crops. It is primarily caused by the SBWMV, a member of the genus furovirus within the family Virgaviridae [15,16,17]. The virus is transmitted exclusively via soil, utilizing the root-infecting protist *Polymyxa graminis* as its specific vector. *P. graminis* produces motile zoospores that penetrate wheat root hairs and release the virus intracellularly. Once inside the host, the virus spreads systemically and may persist in the soil for years in the absence of crops due to the longevity of *P. graminis* resting spores [18,19]. Unlike many plant viruses, SBWMV is not seed-borne or insect-borne, which poses significant challenges for its control and management.

WSBM symptoms typically include mosaic mottling, chlorotic striping, stunting, and reduced tillering, most visibly during late winter to early spring after plants break dormancy. Symptom expression is highly influenced by environmental conditions such as soil temperature, moisture, and host susceptibility. Infections generally begin in autumn but remain latent for months, further complicating early detection [20,21]. The disease primarily affects *Triticum aestivum* (common wheat), although some isolates may also infect barley and related cereals. It is widely distributed across temperate wheat-producing regions, with outbreaks reported in the United States (Midwest and Northeast), India, China, Japan, and parts of South America [22]. Notably, in regions like New York State, SBWMV infections have caused serious impacts on wheat yield, highlighting its growing threat to global food security. The disease’s expansion in recent years is closely linked to changing cropping practices, climate-induced soil conditions, and widespread use of susceptible cultivars.

**Figure 1:**
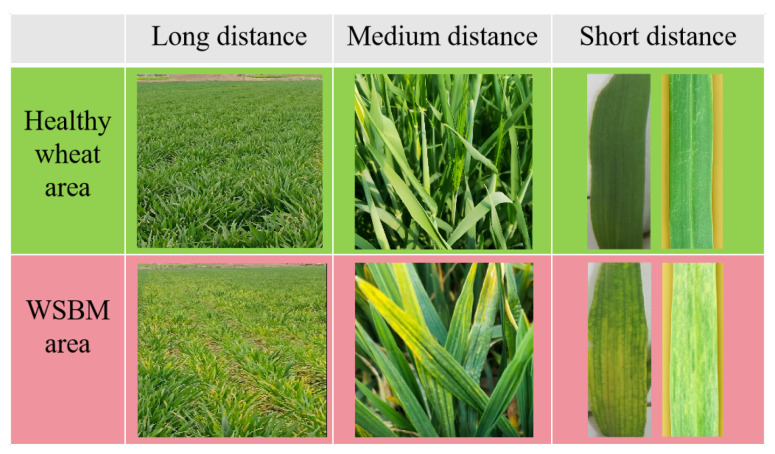
Healthy wheat and wheat infected with SBWMV at different distances from the infection source.

SBWMV poses a major agronomic threat, with yield losses reaching up to 50% in heavily infected fields [23]. Figure 1 illustrates the striking contrast between healthy and infected wheat, where diseased leaves exhibit distinct yellow streaks and geometric mosaic patterns. Because of its patchy field distribution and dependency on fluctuating environmental factors, visual diagnosis alone is often unreliable. In the absence of curative treatments, current management relies on the deployment of resistant cultivars, crop rotation, and adjusted planting schedules. Traditional assessment methods, however, are often inadequate in coping with the complex interplay of multi-regional conditions and dynamically evolving symptoms. This underscores the need for multi-attribute, geographically distributed evaluation systems capable of resolving conflicting assessments and supporting more precise disease management strategies.

**Figure 2:**
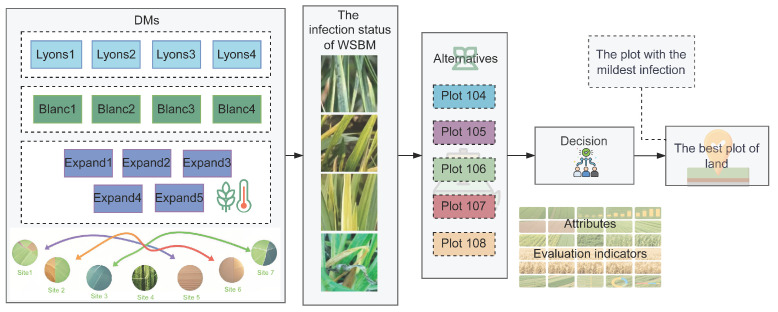
A conceptual architecture of the LSGDM-based WSBM assessment model.

Given the complex interplay between environmental conditions and cultivar susceptibility, WSBM symptom expression and progression often vary across planting sites. Consequently, evaluations of disease severity can differ among geographic locations and expert assessors, reflecting both ecological heterogeneity and subjectivity in evaluations. This study frames the WSBM severity assessment task as a group decision-making problem, wherein each planting plot, based on site-specific observations and ecological context, is treated as a virtual decision maker (DM). Figure 2 visually illustrates how these virtual DMs operate, each contributing a localized evaluation matrix grounded in field-recorded symptoms, mapping out the flow from data collection to the final decision-making process for WSBM assessment. Specifically, given the diversity and number of participating plots—each representing a unique ecological niche and evaluative perspective—this problem conforms to the structure of a large-scale group decision-making (LSGDM) scenario. In this model, (1) rows represent different planting plots (alternatives); (2) columns represent evaluation indicators (e.g., virus load, symptom intensity); (3) expert assessments are modeled as opinions sourced from different plots or ecological subregions; and (4) conflicts arise from inconsistencies in these assessments due to environmental variability and the wheat growth and yield indicator data. By modeling WSBM evaluation as a large-scale, geographically distributed group decision process, this study aims to capture ecological heterogeneity, resolve inter-site opinion divergence, and support trustworthy, data-driven agricultural interventions.

In the following sections, this study introduces the background and motivation for addressing wheat disease assessment using the proposed decision-making methods. Section 1.1 reviews the historical context and recent advancements in plants with AI technologies. Section 1.2 discusses the limitations of traditional decision-making models and presents the theoretical basis for applying LSGDM to agricultural problems. Section 1.3 provides a summary of the innovative aspects and key contributions of the proposed method.

### 1.1. The Historical Context and Recent Advancements in Plants

The monitoring and management of plant diseases and pests have long been central challenges in agricultural production. In recent years, the rapid development of AI has led to significant progress in this field, particularly through the application of computer vision and deep learning technologies [24,25,26]. These approaches have been widely adopted to improve the early detection of symptoms and facilitate targeted responses.

Azizi et al. [27] developed a multi-task model combining UAV-captured RGB images, ResNet50, EfficientNet-B7, and LSTM, with an improved loss function to address data imbalance, achieving over 91% accuracy in detecting the wheat lodging’s ratio, angle, and position. Devran and Goknur [28] developed a system capable of quickly and accurately identifying NRKs for root-knot nematodes, making it suitable for use in various laboratory environments. Gautam et al. [29] used drone imagery and the YOLOv5 deep learning model to study the flowering stage detection of the invasive species *Chromolaena odorata* in Queensland, Australia, proposing a low-cost, high-precision automated monitoring method to replace traditional manual surveys. These studies reflect the growing maturity of AI in visual recognition, trait extraction, and phenotype identification [30,31]. However, they primarily focus on symptom detection and classification tasks and often fall short when applied to complex decision-making scenarios—especially those involving expert reasoning, spatial heterogeneity, and the integration of conflicting assessments [32,33].

While image-based models excel at visual pattern recognition, they lack the capacity to integrate distributed expert knowledge, resolve inter-site inconsistencies, or support strategic decision making under uncertainty. In contrast to image-driven approaches, this study addresses the problem of disease evaluation from a decision-making perspective [34]. Specifically, this study frames the assessment of WSBM severity as a LSGDM process, where each planting site acts as a virtual DM contributing context-specific judgments. This decision-based modeling structure is designed to reconcile inconsistencies across expert assessments and ecological conditions, supporting a more interpretable, scalable, and trust-aware framework for plant disease management.

### 1.2. LSGDM Models with Enhanced Spectral Clustering and Decision Indicator Sets

Assessing the severity of SBWMV infection is inherently complex, requiring the integration of diverse data sources, expert knowledge, and often conflicting perspectives [20,35]. Traditional individual-based decision-making approaches are frequently inadequate in this context, as they lack the capacity to comprehensively consider the multi-faceted and interdisciplinary nature of agricultural disease evaluation. These methods often fall short in accommodating the variability in expert opinions, the uncertainty of environmental conditions, and the dynamic interactions among factors.

To address these limitations, LSGDM methods have emerged as a promising solution. These approaches facilitate a more holistic and systematic evaluation by synthesizing the judgments, preferences, and domain expertise of a broad panel of DMs [36,37,38]. By leveraging collective intelligence, LSGDM enables more robust, transparent, and scientifically grounded decision outcomes that are better suited to real-world agricultural challenges. In recent years, LSGDM has garnered significant attention in decision science research, with scholars advancing the methodology from various perspectives. For instance, Li et al. [39] developed a group decision-making approach that balances consistency and formalization, while Yu et al. [40] proposed a rough-set-based group-oriented superiority relation method to address complex problems involving a large number of DMs. These contributions have significantly propelled the development of LSGDM.

However, practical implementation still faces challenges, such as handling vague evaluation information provided by DMs and achieving group consensus [41,42]. To bridge the gap between theory and practical implementation, this study proposes a novel group decision-making framework that incorporates AI techniques, trust-weighted opinion modeling, and consensus reaching process (CRP) mechanisms. The model is specifically designed to support plant disease assessment in large-scale contexts like WSBM prevention and control.

The framework introduces a CRP based on enhanced spectral clustering aimed at identifying opinion clusters, resolving inconsistencies, and optimizing group consensus. It further integrates trust mechanisms and MG approximation to rank intervention strategies more objectively. Through a combination of personalized similarity metrics, feedback-based preference revision, and multi-stage subgroup formation, the model strives to balance scientific rigor and practical usability.

### 1.3. The Summary of Research Challenges and Motivations

To overcome the complex challenges posed by cross-regional WSBM evaluation, such as symptom heterogeneity, inconsistent expert judgment, and ecological uncertainty, this study proposes a CRP model that integrates AI and decision science methodologies within the LSGDM framework. The model is built on enhanced spectral clustering techniques and a multi-stage feedback mechanism, enabling a more accurate and efficient evaluation of SBWMV infection severity. Specifically, this study aims to resolve the following key issues:

(1) How to address the ambiguity in field assessments caused by evaluator subjectivity, environmental noise, and symptom overlap with non-disease factors.
(2) How to manage inconsistent evaluations and estimate missing trust relationships among geographically dispersed planting sites.
(3) How to reduce decision complexity and effectively identify opinion divergence within ecologically similar subgroups.
(4) How to structure the group decision-making process to ensure convergence, credibility, and interpretability of the final outcome.

The core idea is to integrate multi-dimensional clustering, trust modeling, and rough set theory to support multi-attribute evaluation and iterative opinion adjustment. The proposed solution consists of the following four keys:

(1) Field-collected WSBM symptom data are encoded using IFNs to represent degrees of infection belief, skepticism, and hesitation, effectively capturing observation ambiguity and assessment uncertainty.
(2) A trust network is constructed based on ecological similarity and spatial proximity. Missing or weak trust values are inferred via graph reasoning to ensure that regional influence and consistency are embedded in the evaluation process.
(3) Combining numerical deviation and rank order differences, a customized distance metric is used to identify subgroups of plots with similar evaluation behaviors. This reduces complexity while preserving ecological interpretability.
(4) ADISs derived from agricultural disease metrics guide iterative revisions of plot-level assessments until opinion differences are reconciled and consensus is achieved. Finally, using ADISs based on MGRS, the consensus matrix is processed to generate robust, explainable rankings of planting plots. This enables targeted interventions, such as deploying resistant varieties or applying localized treatments.

By embedding these techniques within a unified framework, the proposed model not only improves the efficiency and reliability of consensus formation but also enhances the scientific validity and interpretability of the final decisions. The model is validated in a real-world WSBM prevention scenario, and comparative experiments demonstrate its superiority over traditional consensus models in terms of convergence speed and robustness.

The remainder of this study is organized as follows. Section 2 presents the foundational materials and methodologies, including the dataset with its acquisition, preprocessing, and transformation into intuitionistic fuzzy numbers (IFNs), along with core technical frameworks such as spatial trust propagation, spectral clustering, weight calculation, consensus measurement, and feedback-driven convergence. Section 3 demonstrates the practical application of the proposed framework through a real-world case study on WSBM severity evaluation. Section 4 provides additional analyses, including sensitivity studies and comparative evaluations with existing models. Finally, Section 5 concludes the study by summarizing key findings and outlining future research directions. Appendix A, Appendix B and Appendix C, containing supplementary details, are available at the end of this manuscript.

## 2. Materials and Methods

This section elaborates on the foundational data and methodological framework employed to address the challenges of WSBM severity assessment. It first describes the dataset used in the study, including its acquisition, preprocessing, and transformation into a format suitable for uncertainty modeling. Subsequently, it details the core methodologies, covering trust propagation mechanisms, similarity analysis and clustering of planting sites, weight calculation frameworks, consensus measurement, and feedback-driven consensus convergence. These components collectively form the intelligent decision-making framework tailored to cross-regional WSBM evaluation.

### 2.1. Datasets

This subsection introduces the source, characteristics, and preprocessing of the dataset, laying the groundwork for subsequent modeling and analysis.

#### 2.1.1. Data Acquisition and Background

The dataset used in this study was derived from a multi-year field survey and yield monitoring program aimed at characterizing the distribution and impact of SBWMV in the dryland wheat production zones of the U.S.A. Pacific Northwest, particularly in the Walla Walla Valley, which spans the Oregon–Washington state border.

Field data were collected from 2017 and 2018 growing seasons, involving both symptomatic and asymptomatic plots of commercial winter wheat fields planted with a susceptible variety (UI Magic). SBWMV infection was confirmed through RT-PCR molecular detection in tissue samples collected at peak symptom expression (April) [20]. The field plots were geo-referenced and stratified based on symptom presence, allowing for direct comparisons of yield performance across infected and healthy subregions.

In total, 24 field plots (12 symptomatic, 12 asymptomatic) were monitored per year. Data recorded included the following:

- Grain yield (kg/ha): Core for measuring wheat production. High yield means efficient conversion of resources to grains; low yield relates to poor pollination, affecting profits and supply.
- Above-ground biomass (kg/m^2^): Reflects photosynthetic capacity. More biomass supports grain development; less (due to stress) limits yield and reduces stress resistance.
- Wheat head count (heads/m^2^): A basic yield-structuring index. Proper count (via density/management) balances population and individual growth for more grains.
- Spikelets per head: Determines potential grains per ear. Affected by genetics and environment; more spikelets (with good conditions) boost yield potential.
- Test weight (kg/l): Shows grain quality/fullness. Higher weight means better quality (more starch, good for milling); lower weight signals poor grain development from stress, linking to yield/quality.

**Figure 3:**
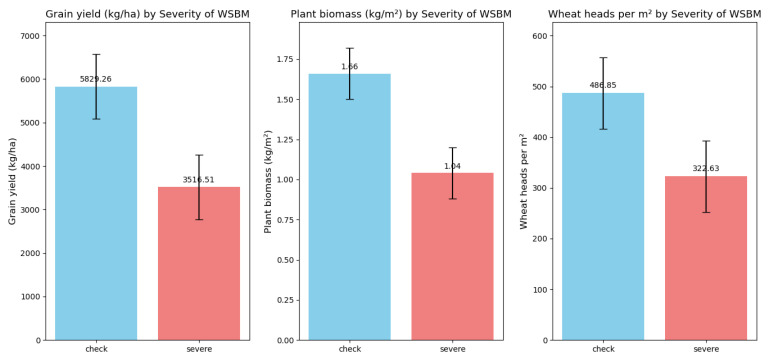
The impact of SBWMV infection severity across key growth parameters.

These metrics—grain yield, above-ground biomass, wheat head count, spikelets per head, and test weight—collectively gauge wheat growth vitality, yield potential, and grain quality, and these growth indicators will serve as decision-making metrics. The comparison chart of the impact of SWBMV infection severity on winter wheat yield concerning the above growth parameters is shown as Figure 3.

This dataset reflects a complex interaction of environmental conditions (soil moisture, temperature) and plant health metrics, making it suitable for evaluating decision performance under ecological uncertainty and field-level variability, which are central challenges in WSBM severity assessment.

#### 2.1.2. Data Preprocessing and Transformation into Intuitionistic Fuzzy Numbers

Given that the original dataset contained only two years of field data (2017 and 2018), this study applied a controlled data augmentation strategy solely for validation purposes. Specifically, a 10-year extended dataset was constructed by replicating and perturbing the original samples with ecologically plausible variations (e.g., soil moisture fluctuations, symptom onset timing) while strictly preserving the unique ecological, technical, and managerial characteristics of each planting site. This process does not homogenize site-specific data or override inter-site reliability differences. Instead, it enables temporal robustness tests without interfering with the trust-based modeling of cross-regional uncertainty. Subsequently, this study performs DM filtering to remove noisy or inconsistent evaluators, resulting in 13 representative DMs retained for modeling.

To effectively handle the uncertainty and ecological ambiguity inherent in WSBM symptom assessment, all normalized indicator data were transformed into IFNs [43]. For each observation $x_{ij}$ from DM $s_{i}$ on indicator $a_{j}$, a fuzzy representation was constructed using the following normalization formula as Equation (1) [44]:

(1) $$x_{\mathrm{norm}}=\frac{x-x_{\min}}{x_{\max}-x_{\min}}$$

Based on the normalized value $x_{\mathrm{norm}}$, the membership degree $\mu_{ij}$ is set to $x_{\mathrm{norm}}$. The non-membership degree is calculated using Equation (2) [44]:

(2) $$v_{ij}=1-\mu_{ij}$$

This IFN representation enables a more flexible and informative encoding of both confidence and uncertainty in disease severity assessments. It provides a robust foundation for the LSGDM process and supports downstream consensus analysis across diverse and ecologically heterogeneous field plots.

### 2.2. Methodology

**Figure 4:**
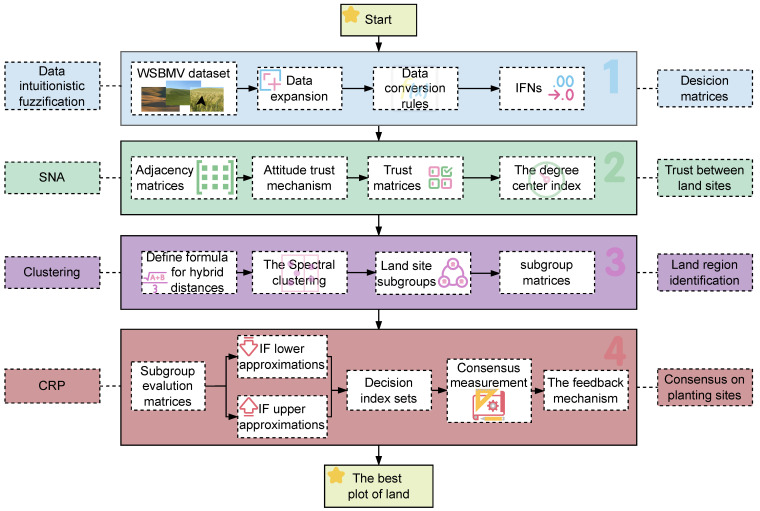
A operational workflow of the LSGDM framework for WSBM consensus detection.

This subsection outlines the technical approaches designed to handle the complexities of LSGDM in WSBM assessment. It encompasses trust modeling, clustering, weight calculation, consensus measurement, and feedback mechanisms, forming a comprehensive pipeline for data-driven agricultural decision making. The overall framework is shown in the Figure 4.

#### 2.2.1. Trust Propagation Analysis of Planting Sites Based on Bayesian Graph Neural Model

In the multi-regional collaborative evaluation scenario of wheat planting, the exchange of information and mutual trust between different planting sites is crucial for comprehensively assessing wheat growth and yield conditions. Due to differences in environmental conditions, planting techniques, variant/cultivar, fertilizations, and management levels at each location, the reliability of the wheat growth and yield indicator data provided also varies [45,46]. For instance, some planting locations may have more reliable data due to accumulated planting experience and good management practices, while others may face instability or reduced reliability of their data due to new planting technologies or unforeseen natural disasters.

**Figure 5:**
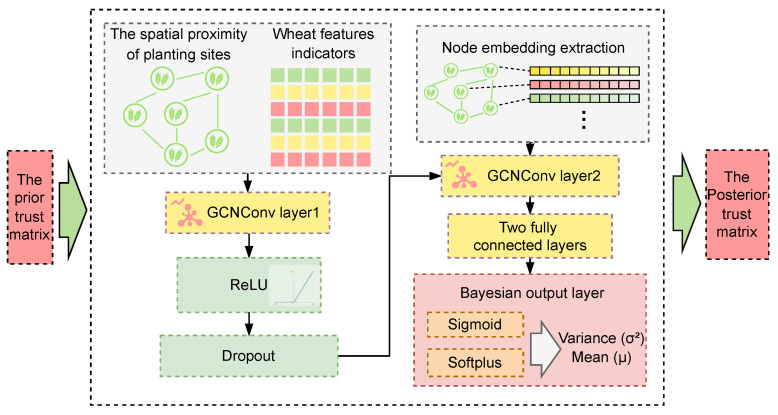
The Bayesian-GCN frameworks for inferring trust in spatial agronomic networks.

Therefore, establishing a trust propagation mechanism between planting locations can better integrate the data from each location, improving the overall evaluation accuracy. The specific propagation process is shown in Figure 5.

A graph structure is constructed with planting locations as nodes, where the features of each node include wheat growth and yield indicators. Bayesian methods are used to model the trust relationships between nodes, representing the degree of trust each node has in the data from other nodes, and directed weighted edges are constructed based on spatial proximity (geographical distance threshold) and functional similarity (ecological indicator similarity). The environmental association logic in the expert assessment process is translated into network trust propagation weights. Hierarchical GCNs are used to enable distributed learning of multi-order spatial dependence features, while Bayesian probability inference is employed to quantify uncertainty in the trust propagation path. Ultimately, a probabilistic trust propagation mechanism is constructed, outputting a predicted distribution of missing trust values for plots, with the mean representing trust strength and the variance reflecting assessment confidence.

The model takes into account the spatial relationships between planting locations (e.g., neighboring locations may share similar environmental conditions, leading to higher trust) and the consistency of ecological indicator similarity. GCNs are used to capture the complex interactions and trust propagation processes between nodes, learning the weights of each node in the social networks (SNs).

#### 2.2.2. The Similarity Analysis and Grouping of Planting Sites Based on Wheat Growth and Yield Indicators

In agricultural decision making, particularly in cross-regional disease assessments such as the WSBM, significant ecological differences and perceptual biases are often encountered. These differences not only manifest in soil properties, climatic conditions, or local cultivar resistance but also in how various DMs perceive disease symptoms [47,48]. To better handle this multi-dimensional divergence, this study proposes a hybrid distance metric that combines numerical evaluations and ranking preferences. This approach enables more accurate clustering analysis and supports the improvement of consistency in the decision-making process.

Next, this study will delve into how similarity analysis based on wheat growth and yield indicators can be used to assess planting sites and how this analysis can be applied to effective clustering, further supporting the cross-regional assessment of WSBM. In this process, the choice of distance metrics and the integration of opinions among DMs will play a crucial role. Specifically, the ordinal distance and cardinal distance are used to quantify the dissimilarity between planting plots based on normalized wheat growth and yield indicators due to their interpretability and suitability for continuous agricultural variables.

To accurately capture this dual-source divergence, this study adopted a hybrid distance metric that simultaneously considers cardinal differences (numerical evaluations) and ordinal differences (rank preferences). This dual-dimensional approach enables a more context-aware clustering of DMs into subgroups, which is crucial for improving the internal consistency of consensus-reaching processes.

**Definition 1.**  *Let $P_{s_{1}}={\left({p_{ij}}^{s_{1}}\right)}_{n\times m}$ and $P_{s_{2}}={\left({p_{ij}}^{s_{2}}\right)}_{n\times m}$ denote the evaluation matrices provided by two DMs $s_{1},s_{2}$, where $s_{1},s_{2}\in S$. The distance between them is calculated using the Euclidean distance formula as Equation (3) [49]:*

(3) $$d_{1}\left(P_{s_{1}},P_{s_{2}}\right)=\sum_{i=1}^{n}\sum_{j=1}^{m}\sqrt{{\left({p_{ij}}^{s_{1}}-{p_{ij}}^{s_{2}}\right)}^{2}},\forall j\in M,i\in N,s_{1},s_{2}\in G.$$

Prior to defining the Hamming distance, it is imperative to first establish the ranking of alternatives for each DM based on the evaluation matrix.

**Definition 2.**  *Let $P_{k}={\left({p_{ij}}^{k}\right)}_{n\times m}$ be the evaluation matrix provided by the k-th DM, which is the score for the i-th alternative (plot) under the j-th evaluation attribute. Let $w_{j}$ be the weight of the j-th attribute. Then, the aggregated severity score for alternative $x_{i}$ according to DM $s_{k}$ is calculated as Equation (4) [49]:*

(4) $$VP_{k}\left(i\right)=\sum_{j=1}^{m}w_{j}{p_{ij}}^{k},\forall j\in M,i\in N.$$

This value reflects the overall WSBM severity perception for each plot. Sorting the values VP(i) from highest to lowest yields the preference ranking vector for DM $s_{k}$, denoted by $Rs=(r_{1},r_{2},\ldots ,r_{n})$, where $r_{h}$ is the index of the alternative ranked *h*-th in terms of severity.

To facilitate a clearer understanding of the calculation process presented in Definition 2, this study provides an illustrative example below.

**Example 1.**  *Assume a DM assigns weights $W={\left(0.3,0.4,0.3\right)}^{T}$ to three attributes and provides the evaluation matrix*

$$Q=\left[\begin{array}{ccc} 0.6 & 0.5 & 0.4\\ 0.4 & 0.3 & 0.5\\ 0.7 & 0.6 & 0.6 \end{array}\right]$$

*Using Equation (4), the aggregated scores can be obtained as $VP_{s}={\left[0.51,0.41,0.63\right]}^{T}$. Comparing the magnitudes of the elements in $VP_{s}$ results in the ranking $R_{s}=\left[3,1,2\right]$, indicating that the third plot is perceived as the most severely affected, followed by the first and second.*

Once all DMs have generated their respective ranking vectors, this study uses positional Hamming distance to quantify the divergence in their perception of WSBM severity across planting sites. This metric captures how many positions differ between two ranking sequences.

**Definition 3.**  *Let $R_{s_{1}}$ and $R_{s_{1}}$ be the ranking vectors of n alternatives from DM $s_{1}$ and DM $s_{2}$, respectively. The positional Hamming distance between two ranking schemes is defined as Equation (5):*

(5) $$d_{2}\left(R_{s_{1}},R_{s_{2}}\right)=\sum_{h=1}^{n}R_{s_{1}}\left(h\right)⊕R_{s_{2}}\left(h\right),for\;\mathrm{all}\;h\in N,s_{1},s_{2}\in G.$$

*where h denotes the h-th position in the scheme ordering R, and the symbol ⊕ is the mathematical notation for the exclusive OR, indicating whether two characters are identical, with identical values being recorded as 0 and unidentical values being recorded as 1. This metric quantifies the total number of positional mismatches between two ranking schemes.*

**Example 2.**  *Consider two ranking vectors $R_{1}=\left[3,2,1\right],R_{2}=\left[1,2,3\right]$; then, $d_{2}\left(R_{1},R_{2}\right)=3⊕1+2⊕2+1⊕3=1+0+1=2$.*

This component captures the priority divergence in perceived disease severity across regions, helping to reveal different focus areas (e.g., some regions prioritize yield loss, others symptom visibility).

**Definition 4.**  *Let $P_{s_{1}}={\left({p_{ij}}^{s_{1}}\right)}_{n\times m}$ and $P_{s_{2}}={\left({p_{ij}}^{s_{2}}\right)}_{n\times m}$ be the evaluation matrices for any DM $s_{1}$ and DM $s_{2}$, respectively, and let $R_{s_{1}}$ and $R_{s_{2}}$ be the ranking results of alternatives for any DM $s_{1}$ and DM $s_{2}$. The combined similarity measure integrating both evaluation consensus and ranking consistency is defined as Equation (6):*

(6) $$\begin{array}{c} d_{3}\left(P_{s_{1}},P_{s_{2}}\right)=\alpha \left(\frac{1}{n}\sum_{i=1}^{n}R_{s_{1}}\left(i\right)⊕R_{s_{2}}\left(i\right))+\left(1-\alpha \right)(\sum_{i=1}^{n}\sum_{j=1}^{m}\sqrt{{\left({p_{ij}}^{s_{1}}-{p_{ij}}^{s_{2}}\right)}^{2}}\right),\\ for\;\mathrm{all}\;i\in N,\;j\in M,s_{1},s_{2}\in G. \end{array}$$

*where the parameter α is the importance parameter for weighting the two distances and is generally taken to be α=0.5.*

In large-scale, multi-regional WSBM severity assessment, directly aggregating opinions from all planting sites is often inappropriate due to ecological variability and perception gaps. Regional differences in symptom manifestation, influenced by factors such as soil conductivity, rainfall, or cultivar susceptibility, as well as the varying interpretations by local experts, can lead to conflicting assessments and weaken group consensus. To address this, this study proposes using spectral clustering as a tool to partition DMs into ecologically and cognitively coherent subgroups. By leveraging the hybrid distance metric defined in Equation (6), this study constructs an affinity graph that reflects both the numerical proximity and preference alignment between DMs, grouping regions with similar WSBM response characteristics.

In this approach, this study constructs a graph where each node represents a DM corresponding to a specific planting site, and edges are weighted by a Gaussian kernel similarity score based on the hybrid distance between their evaluation matrices. The graph Laplacian is then computed, followed by spectral embedding to extract the inherent clustering structure within the DM network. Finally, K-means clustering is applied to the embedded matrix to group DMs into coherent subgroups that reflect both ecological and evaluation similarities in WSBM severity. The detailed procedure is provided in Algorithm 1.
**Algorithm 1.** 
**The enhanced spectral clustering with subgroup interpretation**
**Input:** 
Data points $D=\{x_{1},x_{2},\ldots ,x_{n}\}$, a kernel bandwidth σ, the number of subgroups *k*
**Output:** 
Subgroup labels $\left\{c_{1},c_{2},\ldots ,c_{n}\right\}$, $c_{i}\in \left\{1,2,\ldots ,k\right\}$
 **Step 1: Construct similarity matrix**
*W* 
**for** 
i=1
 **to** 
*n* **do**
    **for** j=1 **to** *n* **do**
        Compute hybrid distance $d_{ij}$ using Equation (6)
        Compute similarity $W_{ij}\leftarrow \exp\left(-\frac{d_{ij}^{2}}{2\sigma^{2}}\right)$
    **end for**
**end for**
**Step 2: Compute graph Laplacian**
*L*
$D\leftarrow \mathrm{diag}(W\cdot 1_{n})$
$L\leftarrow D^{-1/2}\left(D-W\right)D^{-1/2}$
**Step 3: Eigendecomposition**
Compute eigenvectors and eigenvalues: [U,Λ]←eig(L)
Select top-*k* eigenvectors: $U_{\mathrm{top}}\leftarrow \mathrm{first}\;k\;\mathrm{eigenvectors}\;\mathrm{of}\;U$
**Step 4: Embedding normalization**
**for** 
i=1 
**to** 
*n* 
**do**
    Normalize row the vector $y_{i}\leftarrow \frac{U_{\mathrm{top}}\left[i\right]}{∥U_{\mathrm{top}}\left[i\right]∥}$
**end for**
**Step 5: K-means clustering**
$\left\{c_{1},c_{2},\ldots ,c_{n}\right\}\leftarrow \mathrm{KMeans}\left(\left\{y_{1},y_{2},\ldots ,y_{n}\right\},k\right)$
**return** Subgroup labels $\left\{c_{i}\right\}$


In summary, enhanced spectral clustering not only improves the scalability of the WSBM severity decision model but also respects the regional specificity of agricultural disease expression, making it a biologically meaningful tool for complex consensus formation.

#### 2.2.3. The Weight Calculation Framework

In agricultural LSGDM, traditional weight calculation based solely on the majority principle (subgroup size) overlooks critical factors like trustworthiness and ecological relevance. To address this, this study introduces a trust-centric weight determination framework that integrates SNA to quantify subgroup influence and credibility [41].

**Definition 5.**  *Trust relationships among planting sites are modeled via a fuzzy social trust matrix $T={\left(t_{kh}\right)}_{d\times d}$, where ${\left(t_{kh}\right)}_{d\times d}\in \left[0,1\right]$ denotes the trust degree from DM $s_{k}$ to $s_{h}$. The degree centrality index $CI_{k}$ is used to measure each DM’s structural importance in the social network, as shown in Equation (7).*

(7) $$CI_{k}=\frac{1}{d-1}\sum_{i=1,j\neq k}^{d}t_{ik}.$$

*The weight of the DM $s_{k}$ is defined as Equation (8):*

(8) $$w_{k}=\frac{CI_{k}}{\sum_{i=1}^{d}CI_{k}}.$$

**Definition 6.**  *The credibility of a subgroup $C_{h}$ is calculated as Equation (9):*

(9) $$TS_{h}=\frac{1}{d-\#C_{h}}\cdot \frac{1}{\#C_{h}}\sum_{i=1,i\neq k}^{d}t_{ik}.$$

*where $\#C_{h}$ denotes the number of DMs in subgroups.*

**Definition 7.**  *To comprehensively reflect both the credibility and the relative size of each subgroup, the decision weight $\lambda_{h}$ for a subgroup $C_{h}$ is computed by integrating its credibility score $TS_{h}$ and cardinality $\#C_{h}$ as Equation (10):*

(10) $$\lambda_{h}=\frac{\#C_{h}}{\sum_{h=1}^{b}\#C_{h}}\cdot \frac{e^{TS_{h}}}{\sum_{h=1}^{b}e^{TS_{h}}}.$$

#### 2.2.4. The Consensus Measurement with ADISs-MGRS

In the cross-regional assessment of WSBM, the ecological heterogeneity of the planting plots (such as soil types and climatic conditions) and the uncertainty of symptom observation have made the consistency quantification of the multi-site assessment matrix a challenge. This study introduces the MGRS theory. By constructing DIS, it analyzes and evaluates differences from multiple granularity levels to achieve scientific measurement and decision support of group consensus.

Let $U=\{x_{1},x_{2},\ldots ,x_{n}\}$ be wheat plots and X⊆U denote minimally infected plots. The multi-granulation space is $\varsigma =\{G_{1},G_{2},\ldots ,G_{m}\}$, where granularities correspond to the wheat growth indicator.

(1) Lower approximation: Plots that are definitely considered severely infected under the subgroup definition, as shown in Equation (11)
  (11) $$\underline{G_{i}}\left(X\right)=\left\{x\in U|{\left[x\right]}_{G_{i}}\subseteq X\right\},$$
  ${\left[x\right]}_{G_{i}}$ is the equivalence class of plot *x* under the index $G_{i}$, where it is required that the plot is judged as the least infected among all similar plots.
(2) Upper approximation: It indicates the existence of similar plots that belong to the least infected category, as shown in Equation (12).
  (12) $$\bar{G_{i}}\left(X\right)=\left\{x\in U|{\left[x\right]}_{G_{i}}\cap X\neq ⌀\right\}.$$

To combine granular opinions across all subgroups $C_{1},C_{2},\ldots C_{b}$, this study adopts two MGRS strategies:

(1) Optimistic MGRSs: In the optimistic MGRS framework, a plot is deemed consensually severe if it appears in the lower or upper approximation of at least one subgroup. This embodies a permissive consensus rule, where granular support from any single subgroup (among *b* subgroups $G_{1},G_{2},\ldots ,G_{b}$) is sufficient to recognize severity. Mathematically, the optimistic membership degrees are calculated as Equations (13) and (14):
  (13) $$\begin{array}{c} \mu_{\underline{\varsigma }}^{O}\left(x_{j}\right)=\frac{1}{m}\sum_{i=1}^{m}I[x_{j}\in \underline{G_{i}}\left(X\right)], \end{array}$$
  (14) $$\begin{array}{c} \mu_{\bar{\varsigma }}^{O}\left(x_{j}\right)=\frac{1}{m}\sum_{i=1}^{m}I[x_{j}\in \bar{G_{i}}\left(X\right)]. \end{array}$$
  where the indicator function I marks membership (1) or non-membership (0) in these approximations, and averaging across subgroups quantifies the fraction of subgroups endorsing the plot’s severity (definite or possible).
(2) Pessimistic MGRSs: A plot is deemed consensually severe under the pessimistic MGRS strategy only if it is present in the lower (or upper) approximation of all subgroups simultaneously. This reflects a strict consensus criterion, where a plot’s severity must be consistently recognized across every subgroup’s granular perspective. Mathematically, the pessimistic membership functions are defined as Equations (15) and (16):
  (15) $$\begin{array}{c} \phi_{\underset{-}{\varsigma }}^{P}\left(x_{j}\right)=\frac{1}{m}\sum_{i=1}^{m}I[x_{j}\in \overset{m}{\underset{k=1}{⋂}}\underline{G_{k}}\left(X\right)], \end{array}$$
  (16) $$\begin{array}{c} \phi_{\bar{\varsigma }}^{P}\left(x_{j}\right)=\frac{1}{m}\sum_{i=1}^{m}I[x_{j}\in \overset{m}{\underset{k=1}{⋂}}\bar{G_{k}}\left(X\right)]. \end{array}$$

By combining these two strategies, the framework balances inclusivity (optimistic, capturing diverse granular insights) and rigor (pessimistic, ensuring cross-granular consistency). This dual approach accommodates varying levels of consensus in MG decision making, making it suitable for scenarios like WSBM severity assessment—where some subgroups (e.g., regions with distinct ecological conditions) may have conflicting but valid perspectives. The membership functions $\phi_{\bar{\xi }}^{P}/\phi_{\underline{\xi }}^{P}$ and $\mu_{\bar{\xi }}^{O}/\mu_{\underline{\xi }}^{O}$ quantify the degree of pessimistic/optimistic consensus, enabling systematic fusion of granular opinions while addressing uncertainty and heterogeneity in LSGDM.

By fusing optimistic and pessimistic approximations, three types of DIS are constructed to quantify consensus at different granularity levels:

(1) Optimistic DIS:
  (17) $$T_{1}=\left\{\left.j|\underset{x_{j}\in X}{\arg\max}\;\left\{\phi_{\underset{-}{\varsigma }}^{O}\left(x_{j}\right)+\phi_{\bar{\varsigma }}^{O}\left(x_{j}\right)\right\}\right\}\right.;$$
(2) Pessimistic DIS:
  (18) $$T_{2}=\left\{\left.k|\underset{x_{k}\in X}{\arg\max}\;\left\{\phi_{\underset{-}{\varsigma }}^{P}\left(x_{j}\right)+\phi_{\bar{\varsigma }}^{P}\left(x_{j}\right)\right\}\right\}\right.;$$
(3) Conprehensive DIS:
  (19) $$T_{3}=\left\{\left.l|\underset{x_{l}\in X}{\arg\max}\;\left\{\left\{\phi_{\underset{-}{\varsigma }}^{O}\left(x_{l}\right)+\phi_{\bar{\varsigma }}^{O}\left(x_{l}\right)\right\}⊕\left\{\phi_{\underset{-}{\varsigma }}^{P}\left(x_{l}\right)+\phi_{\bar{\varsigma }}^{P}\left(x_{l}\right)\right\}\right\}\;\right\}\right..$$

The T1 identifies plots with at least one subgroup’s endorsement, enabling adaptive risk exploration in agricultural contexts. The T2 flags plots that are universally supported by all subgroups, driving decisive, high-confidence interventions. The T3 integrates both, prioritizing plots with cross-granular synergy—strong consensus with minimal dissent. Together, T1 fosters early, risk-tolerant detection, T2 ensures rigorous, unanimous action, and T3 delivers holistic, evidence-aligned decision making. This framework systematically quantifies multi-granular agreement, resolving ambiguity in agricultural decision making (e.g., WSBM management) by balancing inclusivity (T1), rigor (T2), and resilience (T3). It aligns granular fusion with practical disease management needs, ensuring resource-efficient, robust, and evidence-based outcomes, critical for addressing ecological heterogeneity and uncertain symptom assessments in agricultural systems.

A consensus judgment framework is established based on the intersection of the three DISs:

(1) **Full consensus:**  When $T_{1}\cap T_{2}\cap T_{3}\neq ⌀$, it indicates consistent evaluation across granularity levels, and plot risk rankings can be directly generated;
(2) **Partial consensus:** If $T_{1}\cap T_{2}\cap T_{3}=⌀$ and $T_{1}\cap T_{2}\neq ⌀$ both hold, feedback adjustment mechanisms must be initiated for divergent indicators;
(3) **No consensus:** When $T_{1}\cap T_{2}=⌀$, systematic divergence is identified, so the feedback adjustment mechanisms must be initiated.

By quantifying consensus at MG levels, the DIS framework resolves ambiguities in agricultural LSGDM, ensuring interventions are tailored to ecological heterogeneity and grounded in rigorous uncertainty modeling via intuitionistic fuzzy operations. This approach directly addresses the challenges of WSBM severity assessment, where symptom variability and ecological diversity demand both inclusive and strict consensus measures, ultimately enhancing the precision and reliability of disease management strategies.

#### 2.2.5. Feedback-Driven Preference Evolution and Consensus Convergence

To reconcile divergent disease severity assessments among geographically distributed wheat plots, especially under varying ecological conditions and symptom expressions, this study designed a dual-channel feedback optimization mechanism for preference evolution and consensus convergence. This mechanism incorporates both subgroup weight calibration and evaluation value adjustment embedded in an iterative decision framework tailored to WSBM assessment. The overall objective is to minimize subgroup disagreement and drive convergence based on multi-granular consensus indicators.

**Step 1. The trust–prospect–entropy-based subgroup reweighting**

Each subgroup $C_{h}$ represents a cluster of planting plots with similar assessment behaviors. This study defines a hybrid update mechanism that accounts for ecological trustworthiness, subjective gain/loss perceptions, and entropy regularization. Let $\mu_{ij}^{h}$ and $v_{ij}^{h}$ denote the membership and non-membership degrees of indicator $a_{j}$ on plot $x_{i}$ within subgroup $C_{h}$, and let $B_{j}=\left(\mu_{j}^{B},v_{j}^{B}\right)$ represent the target values.

First, the deviation of subgroup *h* is computed by comparing its evaluation to the average using Equations (20) and (21):

(20) $${\kappa_{ij}}^{h}=\frac{1}{n}\sum_{k=1}^{n}\frac{{\mu_{ik}}^{h}+{v_{ik}}^{h}}{2},$$

(21) $${\theta_{ij}}^{h}=\frac{{\mu_{ij}}^{h}+{v_{ij}}^{h}}{2}-\kappa_{ij}^{h}.$$

This deviation is transformed using prospect theory into a perceived utility value, as shown in Equation (22):

(22) $${v_{ij}}^{h}=\left\{\begin{array}{c} {\left({\theta_{ij}}^{h}\right)}^{\alpha },\;if\;{\theta_{ij}}^{h}\geq 0,\\ -\lambda {\left({\theta_{ij}}^{h}\right)}^{\beta },\;if\;{\theta_{ij}}^{h}\leq 0. \end{array}\right.$$

where α = 0.88, β = 0.92, and λ = 2.25 represent risk and loss aversion parameters.

Summing across all indicators and alternatives yields the subgroup’s total prospect value $V^{h}$, as shown in Equations (23) and (24).

(23) $${R_{ij}}^{h}={\sum_{e\neq h}1-e}^{\left(-\delta \left({\kappa_{ij}}^{e}-{\kappa_{ij}}^{h}\right)\right)},$$

(24) $$V^{h}=\frac{1}{n\times m}\sum_{i=1}^{n}\sum_{k=1}^{m}\left({v_{ik}}^{h}-{R_{ik}}^{h}\right).$$

The updated weight is then computed as Equation (25):

(25) $$\lambda_{h}^{t+1}=\lambda_{h}^{t}+lr\left(V_{h}+TS_{h}\times \left(\frac{\#C_{h}}{N}\right)-\eta \lambda_{h}^{t}\log\lambda_{h}^{t}\right),$$

where

- ${TS}_{h}$ is the trust score of subgroup h from Bayesian-GCN inference;
- $C_{h}$ is the size of subgroup h;
- *N* is the total number of plots;
- lr is the learning rate;
- η is the entropy control parameter.

**Step 2. The gradient optimization of consensus divergence**

To further refine consensus, this study applies a numerical optimization strategy targeting the maximum disagreement captured by the T3 indicator. Our objective is to minimize it.

This study then updates weights using gradient descent, as shown in Equation (26):

(26) $${\lambda_{h}}^{t+1}={\lambda_{h}}^{t}+lr\Delta L\left({\lambda_{h}}^{t}\right).$$

Weights are re-normalized after each update to ensure $\sum \lambda_{h}=1.$

**Step 3. The evaluation matrix adjustment for preference alignment**

If consensus stagnates, this study directly adjusts subgroup evaluation values to reduce divergence from the consensus matrix *B*. For each (*i*, *j*) pair,

(27) $$\mu_{ij}^{h(t+1)}=\mu_{ij}^{\left(h)(t\right)}-\delta \left(\mu_{ij}^{\left(h)(t\right)}-\mu_{j}^{B}\right),$$

(28) $$\nu_{ij}^{\left(h)(t+1\right)}=\nu_{ij}^{\left(h)(t\right)}-\delta \left(\nu_{ij}^{\left(h)(t\right)}-\nu_{j}^{B}\right),$$

where δ is a small adjustment step. This helps reduce rigid discrepancies when weight tuning is insufficient.

This feedback architecture enables robust, interpretable convergence across geographically and ecologically diverse planting plots in WSBM management.

Against the backdrop of addressing WSBM severity assessment challenges, the pseudocode elaborately detailing the entire CRP grounded in MGRS and feedback-driven weight adjustment is vividly presented in Algorithm 2. This algorithm systematically integrates the dynamic calibration of subgroup weights and ADISs-MGRS consensus measurement, offering a robust operational framework for reconciling divergent evaluations across ecologically heterogeneous planting plots in WSBM management.
**Algorithm 2** 
**The CRP method based on ADISs-MGRS and feedback-driven weight adjustment**
**Input:** Subgroup partitions $\left\{C_{h}\right\}$, the initial subgroup weights $\left\{\lambda_{h}^{0}\right\}$, the initial target matrix $B_{0}$
**Output:** 
The final ranking result $X_{\mathrm{finally}}$ 
**Step 1: The consensus measurement via ADISs-MGRS**
**repeat**
    Compute optimistic consensus $T_{1}$ using Equation (17)
    Compute pessimistic consensus $T_{2}$ using Equation (18)
    Compute comprehensive consensus $T_{3}$ using Equation (19)
    Check convergence condition: whether $T_{1}\cap T_{2}\cap T_{3}\neq \emptyset$ 
**until** Consensus is reached or maximum iterations exceeded 
**Step 2: Subgroups weight update (trust-prospect-entropy-based)** 
**for** each subgroup $C_{h}$ **do**
    Compute reference point $\kappa_{ij}^{h}$ using Equation (20)
    Compute deviation $\theta_{ij}^{h}$ using Equation (21)
    Transform deviation into prospect utility $v_{ij}^{h}$ using Equation (22)
    Compute adjustment factor $R_{ij}^{h}$ capturing disagreement Using Equation (23)
    Aggregate total utility value $V_{h}$ using Equation (24)
    Update weights $\lambda_{h}^{t+1}$ Using Equation (25)
    Normalize weights: ensure $\sum_{h}\lambda_{h}=1$ 
**end for** 
**Step 3: The evaluation matrix feedback adjustment** 
**if** Consensus stagnates **then**
    **for** each evaluation pair (i,j) **do**
        Update membership using Equation (27)
        Update non-membership Using Equation (28)
    **end for** 
**end if** 
**Step 4: Target matrix evolution** 
Aggregate subgroup matrices using updated weights to obtain consensus matrix *B* 
**Step 5: Output final result** 
Compute comprehensive scores $SC_{i}$ from $T_{3}$ 
**return** The final ranking $X_{\mathrm{finally}}$


## 3. Result

This section presents the outcomes of applying the proposed AI-driven LSGDM framework to evaluate the severity of WSBM across geographically distributed wheat plots. For illustrative purposes, the decision-making process is demonstrated using data from a single representative year. For each year in the dataset, the final ranking of plots is determined using a majority voting approach based on individual decision outcomes.

### 3.1. IFN Conversion: Embedding Diagnostic Uncertainty

The WSBM dataset utilized in this study was derived from the processed dataset mentioned above. The experimental subjects are wheat plants, which include both those infected with the WSBM and healthy ones. During the research process, a representative subset of data, focusing on the disease severity of the infected wheat, was selected for analysis.

Specifically, blocks with the same numbers in the experiments of the expanded five locations were used as alternative schemes, resulting in a total of five alternative schemes numbered 104, 105, 106, 107, and 108. Meanwhile, five key attributes were screened out to construct the attribute set, namely, individual plant weight (bund-wt-kg-m), number of ears per unit area (head-ct-ha), yield per unit area (yield-kg-ha), number of spikelets per ear (spikes-per-head), and ear characteristics (head-weight). On this basis, the dataset was systematically expanded, ultimately forming an analysis sample consisting of 13 DMs.

Based on the above dataset, the decision-making method proposed in this study was applied to analyze the infection status of the SBWMV, aiming to provide a scientific basis for the detection and control of WSBM.

To effectively address the inevitable uncertainties in field observations and measurements, such as errors in data collection, for the values corresponding to the five key attributes of wheat in each plot, they were converted into triplets (μ,v) using Equations (1) and (2). The full results can be found in Appendix A. This process quantifies the cognitive uncertainty of experts, thereby improving the model’s ability to adapt to complex field diagnostics.

### 3.2. The Trust Matrix Completion: Based on Spatial–Ecological Similarity

This subsection describe the estimation of trust scores between geographically separated plots using Bayesian-GCN, embedding both spatial proximity and ecological similarity into the trust matrix.

**Figure 6:**
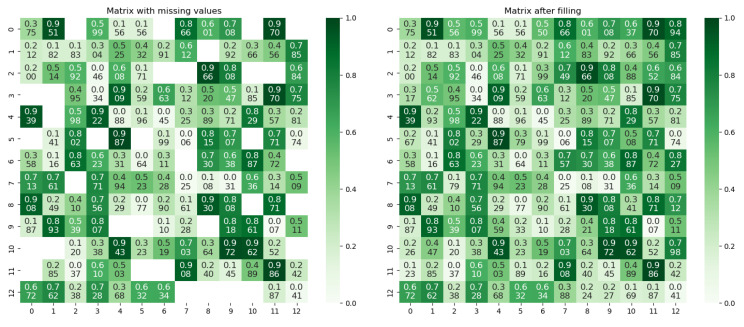
Initial trust matrix and completed trust matrix.

Bayesian-GCN was used to infer missing trust values, constructing a trust network incorporating spatial proximity (e.g., high trust between Lyons 4 and Blanc 1 due to geographic adjacency) and ecological similarity (e.g., higher trust between sites with similar soil pH). Heatmaps in Figure 6 show that the completed trust matrix reflects the feature of “higher intra-ecological zone trust” (e.g., higher trust values within the Lyons group), with parameters detailed in Appendix B. This process mimics agricultural experts’ natural trust in neighboring plots’ data, improving regional evaluation consistency.

### 3.3. The Enhanced Spectral Clustering: Eco-Cognitive Dual-Dimensional Grouping

This process leverages hybrid distance metrics that combine both numerical deviations (for instance, differences in measured virus load or yield loss data) and ranking differences (such as variations in the priority given to symptoms like leaf mosaic versus stunted growth) across evaluation matrices.

Specifically, planting plots with similar wheat growth and yield-related characteristics—including indicators—were grouped into the same subgroup. This grouping is in line with the ecological reality that plots within the same agro-climatic zone (e.g., high-moisture vs. arid regions) or managed with similar wheat cultivars (e.g., resistant vs. susceptible varieties) that usually show consistent WSBM symptom patterns and assessment behaviors.

**Figure 7:**
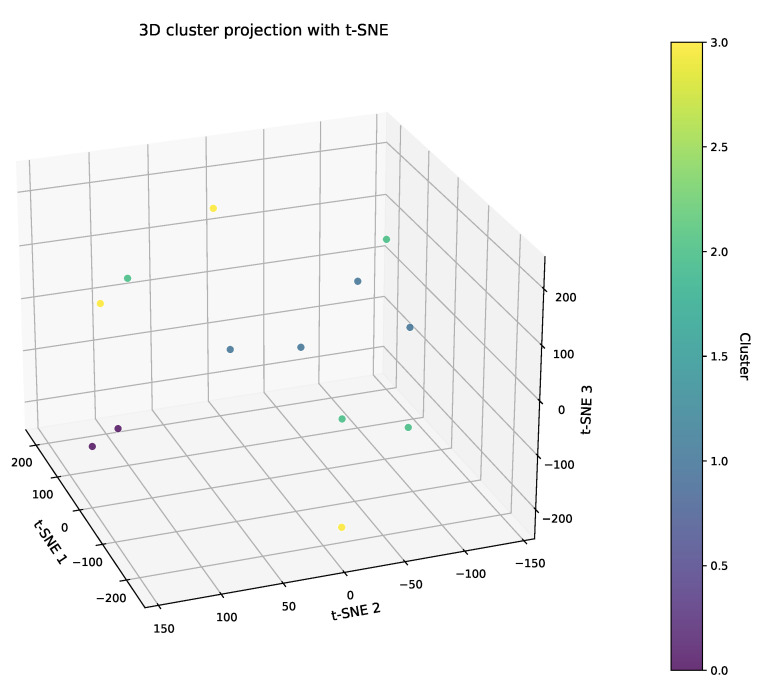
The 3D cluster projection with t-SNE.

The clustering results are presented in two forms. One is the tabular representation (See Table 1), which categorizes 13 relevant elements into distinct subgroups: $G_{0}$ with members $\left\{e_{2},e_{12}\right\}$, $G_{1}$ with $\left\{e_{5},e_{6},e_{7},e_{11}\right\}$, $G_{2}$ with $\left\{e_{0},e_{1},e_{3},e_{4}\right\}$, and $G_{3}$ with $\left\{e_{8},e_{9},e_{10}\right\}$. The other is a 3D scatter plot (as shown in Figure 7). In this plot, each axis represents a principal component. The alignment between these clustering results and the actual field disease distributions, such as the concentration of severe infections in low-lying, waterlogged areas, validates the model’s ability to capture the complex “climate–variety–symptom” associations. This eco-cognitive grouping not only reduces the complexity of decision making but also ensures that the regional specificity in disease expression is retained, providing a biologically meaningful basis for formulating targeted intervention strategies.

### 3.4. The Weight Calculation and Subgroup Aggregation: Incorporating Trust Propagation

For the WSBM dataset’s structural features, this study first calculated each DM weight and subgroup weight. For instance, DMs from plots with denser WSBM symptoms (e.g., severe mosaic patterns in Lyons Block 105) get higher weights via entropy-based data variability analysis.

Next, this study used trust relationships identified in subgroups (e.g., high mutual trust between ecologically similar plots in Lyons and Blanc) to compute inter-subgroup trust correlations. The results (retained to three decimal places, e.g., $G_{0}$’s correlation with $G_{1}=0.823$) are tabulated in Table 2 and Table 3, directly mapping to the dataset’s 13 DMs and four subgroups.

Finally, this study aggregated DMs within each subgroup. This generated subgroup-level evaluation matrices, which aligned with the dataset’s disease severity patterns. Full aggregation details, including raw dataset-to-matrix transformations, can be found in Appendix C.

### 3.5. The Consensus Measurement: Different Subgroup of Planting Sites

This study leveraged the constructed DISs (T1–T3) to quantify evaluation discrepancies across subgroups. As illustrated in Table 4, which maps to subgroup-level assessments within the WSBM dataset, the initial consensus indicators satisfy T1∩T2=⌀. This outcome signals partial conflicts in judging WSBM infection statuses and their impacts across subgroups. Such conflicts stem from variations in experimental conditions (e.g., soil properties, climatic differences between Lyons and Blanc, etc.) and wheat growth performance metrics across subgroups. Activating the feedback mechanism here showcases the model’s capacity to detect cross-subgroup assessment conflicts, a critical capability for precisely deciphering the intricate infection patterns of WSBM within the dataset.

### 3.6. Feedback Regulation: Iterative Optimization of Evaluation Consistency

Subgroup weights and values were dynamically adjusted through trust–prospect–entropy reweighting, incorporating ecological trust scores, perceived utility of assessments, and entropy regularization. Concurrently, divergent evaluations across were fine-tuned iteratively. After 55 adjustment iterations, consensus was achieved. The converged consensus matrix was processed via ADISs-MGRS to generate the final risk rankings of the planting plots: plot104<plot108<plot105<plot107<plot106.

To further validate the temporal robustness and decision consistency of the model, a majority voting strategy was employed across the ten-year augmented dataset. For clarity, each year’s data were independently perturbed but retained the original decision-making context of each planting plot. The consensus-based ranking was computed separately for each year, and the final order was derived by selecting the most frequently occurring rank sequence. This ensemble process reflects natural variations over time without affecting the modeling of cross-regional reliability differences. This ensemble approach accounts for temporal variability and confirms the consistency of decision outcomes. Notably, the aggregated ranking from the 10-year voting procedure was identical to the result from the representative year, reinforcing the model’s temporal stability: plot104<plot108<plot105<plot107<plot106. For instance, plot 105 emerged as the highest-priority intervention site due to its consistently high WSBM severity across all granularity levels, while plot 108 (Blanc Block) was categorized as low risk after feedback alignment. This iterative refinement ensures the model’s outputs align with both data reliability and agricultural decision priorities, providing a actionable foundation for WSBM control strategies.

## 4. Discussions

This section discusses the sensitivity of the model to key parameters, compares its performance with existing methods, and explores the broader value of the plant-based decision modeling approach in agricultural contexts.

### 4.1. Sensitivity Analysis Under Agricultural Heterogeneity

In the context of cross-regional WSBM severity assessment, ecological variation among planting sites, such as differences in soil conductivity, climate conditions, or cultivar susceptibility, introduces uncertainty into expert assessments. As such, verifying the stability and robustness of the proposed CRP under parameter perturbations is essential for real-world agricultural decision making.

This section presents two critical sensitivity analyses aimed at evaluating the influence of model parameters on consensus convergence and ranking outputs.

#### 4.1.1. The Impact of Distance Weighting Parameter α on Plot Clustering Consistency

To capture both cardinal (numerical symptom scores) and ordinal (evaluation rankings) discrepancies among plots, this study adopted a hybrid similarity metric controlled by the distance weighting parameter α. As detailed in Section 2.2.2, α regulates the relative importance of the Hamming distance (rank-based divergence) versus Euclidean distance (value-based divergence).

**Figure 8:**
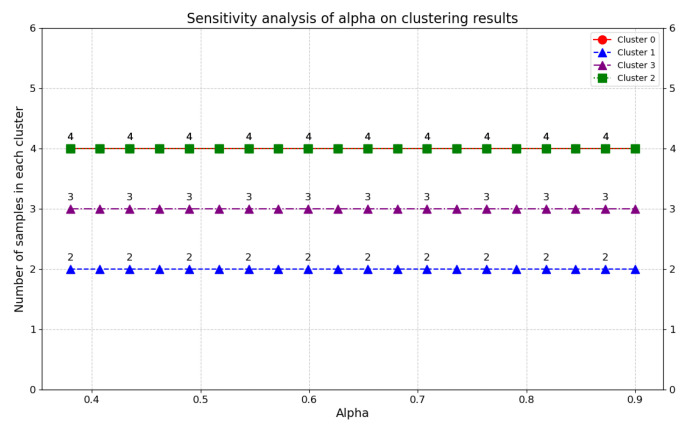
Sensitivity analysis of α on clustering sample distribution.

As illustrated in Figure 8, this study tested values of α ranging from 0.38 to 0.9 and analyzed the number of samples in each cluster across different α settings. Throughout the tested range, the distribution of samples within clusters (Cluster 0 maintained four samples, Cluster 1 had two samples, Cluster 3 held three samples, and Cluster 2 kept four samples) remained extremely stable. Even as α changed, there was no significant fluctuation in the sample count per cluster. This indicates that the model exhibits strong robustness to variations in the distance weighting parameter during the clustering process. Whether emphasizing rank-based Hamming distance or value-based Euclidean distance by adjusting α, the clustering structure of plots stays consistent. It ensures that the interpretability and stability of clustering results are maintained under different weighting preferences, which is crucial for reliable decision-making when prioritizing symptom severity scores or diagnostic rank preferences in WSBM assessment scenarios.

#### 4.1.2. The Effect of Learning Rate (lr) on CRP

**Figure 9:**
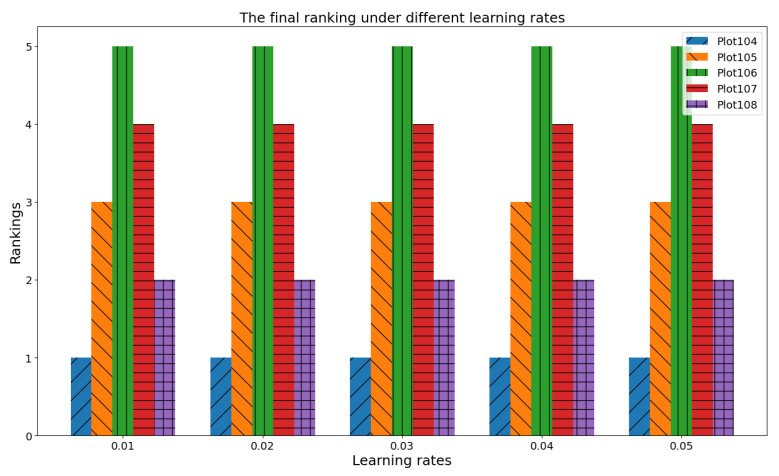
Final ranking variation under diverse learning rates.

The learning rate lr in feedback mechanism governs the step size during weight updates of each subgroup (see Section 2.2.5). A high lr may cause fluctuations and instability, whereas a low value may result in slow convergence. As shown in Figure 9, this experiment varied the lr from 0.01 to 0.05 and observed its impact on the ranking results of different plots (Plot104, Plot105, Plot106, Plot107, and Plot108).

The results reveal that across the tested lr range (0.01–0.05), the relative rankings of the plots exhibited remarkable stability. For instance, Plot104 consistently maintained the highest rank (1), Plot105 stayed at rank 3, Plot106 remained at rank 5, Plot107 held rank 4, and Plot108 was at rank 2. This indicates that trust–prospect–entropy-based optimization module and gradient optimization of consensus endow the consensus convergence process with strong robustness to learning rate variations. Whether in fast-adjusting (higher lr) or slow-adjusting (lower lr) scenarios, the core ranking logic of plots, which is crucial for targeted WSBM management decisions, remains intact, ensuring reliable decision making in dynamic agroecological environments.

### 4.2. Comparison Analysis

In this section, this study conducted comparative experiments to highlight the relative effectiveness of our proposed model. The comparative analysis was divided into two parts. First, this study performed an ablation study by selectively removing or replacing core components of the framework (e.g., clustering, trust propagation, decision indicator sets), which helped assess the individual contribution of each module. Second, this study compared the complete model with baseline GDM methods widely used in agricultural contexts. These comparisons aimed to verify whether integrating spectral clustering, ecological trust modeling, and ADISs-MGRS contributed to improved decision-making reliability, interpretability, and computational efficiency in real-world WSBM detection tasks.

#### 4.2.1. Ablation Study: Role of Key Modules in Agricultural Decision Making

To evaluate the contribution of each individual component in the proposed LSGDM framework for WSBM assessment, this study conducted a systematic ablation study. Six variant models were created by either eliminating or substituting one key element at a time. Table 5 summarizes the comparative methods.

These modifications reflect simplified versions of the model that exclude domain-specific enhancements relevant to agricultural decision making (e.g., region-based trust, priority weighting based on symptom indicators, etc.).

Table 6 presents the computational performance and ranking outcomes of each method variant.

From the comparison, the following can be observed.

Removal of trust propagation (m3) significantly disrupted the final ranking structure. This confirms the importance of ecological–social information in regional disease convergence, as Bayesian-GCN-enabled trust propagation more accurately reflects inter-site reliability under varying field conditions.

Removing ranking or ordinal metrics (m1,m2) led to inconsistencies in plot prioritization, suggesting that accounting for not only numerical values but also perception-based evaluation sequences is vital in agricultural contexts where decision makers differ in field focus.

Substituting DIS with linear fusion (m5) reduced the convergence interpretability and weakened the biological plausibility of the results. ADISs help structure consensus formation around agriculturally meaningful indicators like virus load and symptom progression, which simple averaging cannot achieve.

In summary, the ablation results reinforce the necessity of integrating domain-specific modules into decision frameworks for plant disease assessment. Particularly in WSBM scenarios, where field variability, expert disagreement, and ecological uncertainty are intertwined, each module plays a critical role in enhancing scientific rigor, system robustness, and practical relevance.

#### 4.2.2. Comparative Performance Evaluation with Existing Models

To comprehensively evaluate the performance of the proposed framework in practical agricultural contexts, this study conducted a comparative experiment against three established group decision-making models proposed by Hou et al. [49], Xu et al. [50], and Liu et al. [51] These representative approaches have been applied in multi-attribute group decision studies and serve as robust benchmarks for consensus building and alternative ranking.

All models were tested under the same WSBM severity assessment task using IF data from five key wheat plots across the Lyons and Blanc regions. Each model was evaluated from the perspectives of group structure adaptability, convergence efficiency, and ranking robustness. The results are summarized in Table 7:

Compared with the three models proposed by Hou et al. [49], Xu et al. [50], and Liu et al. [51], the proposed method exhibits advantages in the context of WSBM LSGDM. Most notably, it is the only model that explicitly incorporates ecological and trust-based subgrouping, enabling more accurate reflection of the spatial heterogeneity inherent in agricultural disease assessments. While its convergence speed (10 iterations) is marginally slower than Hou et al. [49]’s model (5 iterations), it surpasses Liu et al. [51]’s in both efficiency and ranking reliability. Furthermore, the proposed method has the highest Kendall rank correlation coefficient, demonstrating superior ranking stability and resistance to noise or evaluation inconsistency. This is particularly critical in real-world agricultural settings, where regional differences in environment, symptom expression, and expert perception frequently lead to divergent evaluations. Overall, these results highlight the model’s strong adaptability, interpretability, and robustness in supporting informed and localized decision-making for complex crop disease scenarios.

### 4.3. Theoretical and Practical Value of Plant-Based Decision Modeling

The plant-based modeling approach proposed in this study redefines the role of individual planting sites within agricultural decision systems. By treating each plot as a virtual DM, the model shifts from traditional expert-centric evaluations toward a decentralized structure where knowledge is embedded at the field level. This theoretical abstraction is particularly well suited for modern agriculture, which is increasingly characterized by spatial fragmentation, diverse cultivation practices, and locally varying ecological conditions.

From a theoretical standpoint, this modeling strategy contributes to the development of distributed decision-making mechanisms under uncertainty. It enables the integration of fuzzy symptom observations, expert perceptions, and environmental heterogeneity within a unified computational framework. The fusion of trust networks, spectral clustering, and rough set-based ranking provides a comprehensive approach for balancing global coherence with local specificity.

Practically, the plant modeling framework enhances agricultural decision-making by enabling region-sensitive interventions. In the context of WSBM assessment for insatnce, it allows for prioritizing plots that not only exhibit severe symptoms but also hold strategic importance in disease spread or yield protection. Moreover, the framework supports modular adaptation, making it applicable to a broad range of agricultural scenarios—from pest outbreak coordination and varietal selection to soil health monitoring and irrigation scheduling.

As agricultural decision making evolves toward multi-source data integration and real-time response, the plant-based modeling philosophy offers a scalable, interpretable, and stakeholder-aligned path forward. It transforms spatial disease data into structured group knowledge, providing a foundation for more transparent, adaptive, and actionable decisions in complex agro-ecological systems.

## 5. Conclusions

This study presents a consensus-reaching framework tailored for evaluating WSBM severity across heterogeneous ecological regions. Recognizing the limitations of traditional expert-driven assessments, namely, subjectivity, regional inconsistency, and lack of scalability, this study reformulates the WSBM evaluation task as an LSGDM problem, where each planting plot acts as a virtual DM. This modeling strategy effectively captures the distributed and data-rich nature of modern agricultural disease surveillance.

To address the challenges of ecological heterogeneity, inter-site trust asymmetry, and evaluation uncertainty, the proposed model integrates multiple AI-enabled modules: a spatial trust propagation mechanism based on ecological similarity and GCNs; a personalized spectral clustering algorithm driven by hybrid distance metrics to group plots with similar patterns; and a feedback-controlled consensus-reaching process guided by ADISs. Final decisions are interpreted using a MGRS model, ensuring robustness across multiple cognitive and data granularity layers.

The above experiments based on real-world WSBM field data demonstrate that the proposed model outperforms existing methods in terms of convergence stability, ranking consistency, and domain interpretability. Sensitivity analyses confirm the robustness of model behavior under different parameter settings, while ablation and comparative studies highlight the individual contributions of each module to overall system performance.

Overall, this study presents an AI-driven decision-making framework that effectively integrates ecological data, expert assessments, and uncertainty modeling to support large-scale plant disease evaluation. Anchored in the case of WSBM, the proposed model demonstrates robust performance in addressing cross-regional variability—such as divergent symptom expressions under different climatic conditions—resolving conflicting assessments from geographically dispersed experts and accommodating ecological heterogeneity in soil properties and crop management practices. Beyond WSBM management, the methodology exhibits strong generalizability to other complex agricultural decision scenarios: it can guide cultivar selection by weighing regional adaptability and disease resistance, enable early warning of pest outbreaks through real-time monitoring of environmental triggers, and optimize resource allocation (e.g., fertilizer or irrigation) under uncertainty by balancing yield goals and risk mitigation.

Future research will advance the framework’s practical applicability through three targeted directions. First, for real-time deployment, this study will integrate edge computing with IoT sensors to enable on-site data processing, reducing latency in disease severity assessment and facilitating immediate management responses (e.g., localized fungicide application). Second, in terms of UAV-based symptom monitoring, this study will develop multi-modal data fusion pipelines that combine high-resolution RGB imagery, multi-spectral data (to capture chlorophyll changes), and thermal imaging (to detect stress-induced temperature variations), enhancing the accuracy of remote symptom recognition and reducing reliance on manual scouting. Third, for adaptive decision updates, this study will implement online learning mechanisms that continuously refine the model using multi-seasonal data streams, incorporating seasonal trends (e.g., rainfall patterns affecting virus transmission) and long-term changes in soil health, ensuring that the framework remains robust across dynamic agricultural environments.

## Figures and Tables

**Table 1:** The subgroup corresponding to planting sites.

Subgroups	Planting Sites
$G_{0}$	$\left\{s_{2},s_{12}\right\}$
$G_{1}$	$\left\{s_{5},s_{6},s_{7},{s_{1}}_{1}\right\}$
$G_{2}$	$\left\{e_{0},e_{1},e_{3},e_{4}\right\}$
$G_{3}$	$\left\{e_{8},e_{9},e_{10}\right\}$

**Table 2:** Each planting site weight.

$s_{1}$	$s_{2}$	$s_{3}$	$s_{4}$	$s_{5}$	$s_{6}$	$s_{7}$
0.068	0.076	0.070	0.085	0.082	0.045	0.058
$s_{8}$	$s_{9}$	$s_{10}$	$s_{11}$	$s_{12}$	$s_{13}$	
0.075	0.084	0.087	0.091	0.090	0.089	

**Table 3:** The subgroup weights.

$G_{h}$	${\mathrm{TS}}_{h}$	$\lambda_{h}$
$G_{0}$	3.402	0.435
$G_{1}$	2.079	0.291
$G_{2}$	2.079	0.173
$G_{3}$	2.495	0.101

**Table 4:** The value of ADISs.

ADISs	Values	Max Triples
T1	$\left[(0,0.096),(1,0.112),(2,0.122),(3,0.097),(4,0.132)\right]$	(4,0.132)
T2	$\left[(0,0.122),(1,0.134),(2,0.182),(3,0.14),(4,0.146)\right]$	(2,0.182)
T3	$\left[(0,0.218),(1,0.245),(2,0.304),(3,0.237),(4,0.277)\right]$	(2,0.304)

**Table 5:** A summary of the required comparison methods for WSBM assessment framework.

Original Method	m0
Method removes the cardinal distance.	m1
Method removes ordinal distance.	m2
Method removes SNA.	m3
Method removes prospect–regret theory.	m4
Method replaces DISs with linear fusion.	m5

**Table 6:** Ranking outcomes and computational metrics of ablation experiment.

Method	Total Time (s)	The Clustering Time (s)	The CRP Time (s)	The Final Ranking
m0	5.282	0.648	0.258	plot104≻plot108≻plot105≻plot107≻plot106
m1	4.973	0.603	0.351	plot104≻plot105≻plot106≻plot107≻plot108
m2	5.186	0.628	0.336	plot104≻plot108≻plot105≻plot106≻plot107
m3	1.184	0.604	0.258	plot106≻plot104≻plot105≻plot107≻plot108
m4	5.105	0.643	0.232	plot104≻plot108≻plot105≻plot107≻plot106
m5	5.410	0.644	0.262	plot104≻plot108≻plot105≻plot107≻plot106

**Table 7:** An analysis comparing our method with other methods.

Methods	Robust Group	Iterations	Final Ranking	Ranking Stability
Hou et al. [49]	No	5	plot104≻plot108≻plot105≻plot107≻plot106	0.976
Xu et al. [50]	No	8	plot104≻plot108≻plot106≻plot107≻plot105	0.792
Liu et al. [51]	No	12	plot105≻plot104≻plot108≻plot107≻plot106	0.853
Our method	Yes	10	plot104≻plot108≻plot105≻plot107≻plot106	1.097

## Data Availability

The data will be made available upon request.

## Appendix A

**The DMs’ evaluation matrices**	
$\left[\begin{array}{ccccc} \left(0.82,0.18\right) & \left(0.64,0.36\right) & \left(0.69,0.31\right) & \left(0.05,0.95\right) & \left(0.36,0.64\right)\\ \left(0.64,0.36\right) & \left(0.68,0.32\right) & \left(0.43,0.57\right) & \left(0.03,0.97\right) & \left(0.25,0.75\right)\\ \left(0.74,0.26\right) & \left(0.68,0.32\right) & \left(0.51,0.49\right) & \left(0.07,0.93\right) & \left(0.40,0.60\right)\\ \left(0.67,0.33\right) & \left(0.68,0.32\right) & \left(0.35,0.65\right) & \left(0.02,0.98\right) & \left(0.21,0.79\right)\\ \left(0.57,0.43\right) & \left(0.48,0.52\right) & \left(0.00,1.00\right) & \left(0.05,0.95\right) & \left(0.31,0.69\right) \end{array}\right]$	$\left[\begin{array}{ccccc} \left(0.78,0.22\right) & \left(0.32,0.68\right) & \left(0.64,0.36\right) & \left(0.04,0.96\right) & \left(0.29,0.71\right)\\ \left(0.85,0.15\right) & \left(0.75,0.25\right) & \left(0.71,0.29\right) & \left(0.06,0.94\right) & \left(0.42,0.58\right)\\ \left(0.42,0.58\right) & \left(0.41,0.59\right) & \left(0.36,0.64\right) & \left(0.08,0.92\right) & \left(0.50,0.50\right)\\ \left(0.52,0.48\right) & \left(0.49,0.51\right) & \left(0.11,0.89\right) & \left(0.01,0.99\right) & \left(0.01,0.99\right)\\ \left(0.95,0.05\right) & \left(1.00,0.00\right) & \left(0.68,0.32\right) & \left(0.04,0.96\right) & \left(0.32,0.68\right) \end{array}\right]$
$\left[\begin{array}{ccccc} \left(0.60,0.40\right) & \left(0.30,0.70\right) & \left(0.82,0.18\right) & \left(0.58,0.42\right) & \left(0.06,0.94\right)\\ \left(0.71,0.29\right) & \left(0.79,0.21\right) & \left(0.31,0.69\right) & \left(0.48,0.53\right) & \left(0.49,0.51\right)\\ \left(0.90,0.10\right) & \left(0.49,0.51\right) & \left(0.27,0.73\right) & \left(0.06,0.95\right) & \left(0.49,0.51\right)\\ \left(0.99,0.01\right) & \left(0.01,0.99\right) & \left(0.06,0.94\right) & \left(0.58,0.43\right) & \left(0.65,0.35\right)\\ \left(0.19,0.81\right) & \left(0.52,0.48\right) & \left(0.23,0.77\right) & \left(0.68,0.32\right) & \left(0.07,0.93\right) \end{array}\right]$	$\left[\begin{array}{ccccc} \left(0.99,0.01\right) & \left(0.68,0.32\right) & \left(0.56,0.44\right) & \left(0.25,0.75\right) & \left(0.83,0.17\right)\\ \left(0.63,0.37\right) & \left(0.62,0.38\right) & \left(0.99,0.01\right) & \left(0.44,0.56\right) & \left(0.30,0.70\right)\\ \left(0.38,0.62\right) & \left(0.79,0.21\right) & \left(0.44,0.56\right) & \left(0.32,0.68\right) & \left(0.65,0.35\right)\\ \left(0.09,0.91\right) & \left(0.54,0.46\right) & \left(0.64,0.36\right) & \left(0.01,0.99\right) & \left(0.22,0.78\right)\\ \left(0.98,0.02\right) & \left(0.83,0.17\right) & \left(0.44,0.56\right) & \left(0.54,0.46\right) & \left(0.92,0.08\right) \end{array}\right]$
$\left[\begin{array}{ccccc} \left(0.66,0.34\right) & \left(0.61,0.39\right) & \left(0.56,0.44\right) & \left(0.09,0.91\right) & \left(0.53,0.47\right)\\ \left(0.95,0.05\right) & \left(0.41,0.60\right) & \left(0.67,0.33\right) & \left(0.32,0.68\right) & \left(0.83,0.18\right)\\ \left(0.62,0.38\right) & \left(0.47,0.53\right) & \left(0.83,0.17\right) & \left(0.40,0.60\right) & \left(0.61,0.39\right)\\ \left(0.56,0.44\right) & \left(0.81,0.20\right) & \left(0.99,0.01\right) & \left(0.01,1.00\right) & \left(0.92,0.08\right)\\ \left(0.49,0.51\right) & \left(0.50,0.50\right) & \left(0.49,0.51\right) & \left(0.01,0.99\right) & \left(0.10,0.90\right) \end{array}\right]$	$\left[\begin{array}{ccccc} \left(0.44,0.56\right) & \left(0.08,0.92\right) & \left(0.78,0.22\right) & \left(0.72,0.28\right) & \left(0.89,0.11\right)\\ \left(0.10,0.90\right) & \left(0.86,0.14\right) & \left(0.70,0.30\right) & \left(0.36,0.65\right) & \left(1.00,0.00\right)\\ \left(0.13,0.88\right) & \left(0.21,0.79\right) & \left(0.12,0.88\right) & \left(0.02,0.98\right) & \left(0.93,0.07\right)\\ \left(0.24,0.76\right) & \left(0.80,0.20\right) & \left(0.61,0.39\right) & \left(0.19,0.81\right) & \left(0.13,0.87\right)\\ \left(0.83,0.17\right) & \left(0.33,0.68\right) & \left(0.42,0.58\right) & \left(0.71,0.29\right) & \left(0.52,0.48\right) \end{array}\right]$
$\left[\begin{array}{ccccc} \left(0.20,0.80\right) & \left(0.83,0.17\right) & \left(1.00,0.00\right) & \left(0.62,0.38\right) & \left(0.00,1.00\right)\\ \left(0.00,1.00\right) & \left(0.12,0.88\right) & \left(0.55,0.45\right) & \left(0.20,0.80\right) & \left(0.55,0.45\right)\\ \left(0.61,0.39\right) & \left(0.56,0.44\right) & \left(0.86,0.14\right) & \left(0.00,1.00\right) & \left(0.86,0.14\right)\\ \left(0.31,0.69\right) & \left(0.88,0.12\right) & \left(0.48,0.52\right) & \left(0.46,0.54\right) & \left(0.16,0.84\right)\\ \left(0.82,0.18\right) & \left(0.23,0.78\right) & \left(0.79,0.21\right) & \left(0.64,0.36\right) & \left(0.78,0.22\right) \end{array}\right]$	$\left[\begin{array}{ccccc} \left(0.13,0.87\right) & \left(0.31,0.69\right) & \left(0.54,0.46\right) & \left(0.22,0.78\right) & \left(0.67,0.33\right)\\ \left(0.06,0.94\right) & \left(0.80,0.20\right) & \left(0.83,0.17\right) & \left(0.20,0.80\right) & \left(0.73,0.27\right)\\ \left(0.17,0.83\right) & \left(0.63,0.37\right) & \left(0.19,0.81\right) & \left(0.63,0.38\right) & \left(0.62,0.38\right)\\ \left(0.87,0.13\right) & \left(0.84,0.16\right) & \left(0.51,0.49\right) & \left(0.48,0.52\right) & \left(0.09,0.91\right)\\ \left(0.21,0.79\right) & \left(0.10,0.90\right) & \left(0.72,0.28\right) & \left(0.23,0.77\right) & \left(0.11,0.89\right) \end{array}\right]$
$\left[\begin{array}{ccccc} \left(0.46,0.54\right) & \left(0.38,0.62\right) & \left(0.19,0.81\right) & \left(0.65,0.35\right) & \left(0.80,0.20\right)\\ \left(0.69,0.31\right) & \left(0.65,0.35\right) & \left(0.13,0.87\right) & \left(0.68,0.32\right) & \left(0.44,0.57\right)\\ \left(0.72,0.28\right) & \left(0.31,0.69\right) & \left(0.29,0.71\right) & \left(0.53,0.47\right) & \left(0.62,0.38\right)\\ \left(0.73,0.27\right) & \left(0.76,0.24\right) & \left(0.07,0.93\right) & \left(0.29,0.71\right) & \left(0.41,0.59\right)\\ \left(0.98,0.02\right) & \left(0.08,0.92\right) & \left(0.57,0.43\right) & \left(0.13,0.87\right) & \left(0.51,0.49\right) \end{array}\right]$	$\left[\begin{array}{ccccc} \left(0.41,0.59\right) & \left(0.22,0.79\right) & \left(0.18,0.82\right) & \left(0.62,0.38\right) & \left(0.89,0.11\right)\\ \left(0.94,0.06\right) & \left(0.18,0.82\right) & \left(0.54,0.46\right) & \left(0.59,0.41\right) & \left(0.40,0.60\right)\\ \left(0.52,0.48\right) & \left(0.42,0.58\right) & \left(0.22,0.78\right) & \left(1.00,0.00\right) & \left(0.84,0.16\right)\\ \left(0.67,0.33\right) & \left(0.67,0.33\right) & \left(0.35,0.65\right) & \left(0.25,0.75\right) & \left(0.22,0.78\right)\\ \left(0.62,0.38\right) & \left(0.55,0.45\right) & \left(0.34,0.66\right) & \left(0.92,0.08\right) & \left(0.69,0.31\right) \end{array}\right]$
$\left[\begin{array}{ccccc} \left(0.44,0.56\right) & \left(0.31,0.69\right) & \left(0.04,0.96\right) & \left(0.52,0.49\right) & \left(0.28,0.72\right)\\ \left(0.59,0.41\right) & \left(0.00,1.00\right) & \left(0.11,0.89\right) & \left(0.58,0.42\right) & \left(0.44,0.57\right)\\ \left(0.91,0.09\right) & \left(0.76,0.24\right) & \left(0.46,0.54\right) & \left(0.80,0.20\right) & \left(0.83,0.17\right)\\ \left(0.67,0.33\right) & \left(0.65,0.35\right) & \left(0.30,0.70\right) & \left(0.69,0.31\right) & \left(0.58,0.42\right)\\ \left(0.51,0.49\right) & \left(0.37,0.63\right) & \left(0.23,0.77\right) & \left(0.41,0.59\right) & \left(0.51,0.49\right) \end{array}\right]$	$\left[\begin{array}{ccccc} \left(0.98,0.02\right) & \left(0.81,0.19\right) & \left(0.77,0.23\right) & \left(0.66,0.34\right) & \left(0.11,0.89\right)\\ \left(0.27,0.73\right) & \left(0.59,0.41\right) & \left(0.77,0.23\right) & \left(0.03,0.97\right) & \left(0.66,0.34\right)\\ \left(0.53,0.47\right) & \left(0.31,0.69\right) & \left(0.05,0.95\right) & \left(0.01,0.99\right) & \left(0.96,0.04\right)\\ \left(0.76,0.24\right) & \left(0.59,0.41\right) & \left(0.38,0.62\right) & \left(0.54,0.46\right) & \left(0.47,0.53\right)\\ \left(0.79,0.21\right) & \left(0.67,0.33\right) & \left(0.36,0.64\right) & \left(0.55,0.45\right) & \left(0.40,0.60\right) \end{array}\right]$
$\left[\begin{array}{ccccc} \left(0.18,0.82\right) & \left(0.25,0.75\right) & \left(0.15,0.85\right) & \left(0.53,0.47\right) & \left(0.00,1.00\right)\\ \left(0.84,0.16\right) & \left(0.76,0.24\right) & \left(0.48,0.52\right) & \left(0.33,0.67\right) & \left(0.40,0.60\right)\\ \left(1.00,0.00\right) & \left(0.48,0.53\right) & \left(0.28,0.72\right) & \left(0.57,0.43\right) & \left(0.58,0.42\right)\\ \left(0.26,0.74\right) & \left(0.14,0.86\right) & \left(0.97,0.03\right) & \left(0.27,0.73\right) & \left(0.86,0.14\right)\\ \left(0.50,0.50\right) & \left(0.33,0.67\right) & \left(0.38,0.62\right) & \left(0.73,0.27\right) & \left(0.18,0.82\right) \end{array}\right]$

## Appendix B

**The matrix with missing values**
$\left(\begin{array}{ccccccccccccc} 0.375 & 0.951 & \mathrm{nan} & 0.599 & 0.156 & 0.156 & \mathrm{nan} & 0.866 & 0.601 & 0.708 & \mathrm{nan} & 0.970 & \mathrm{nan}\\ 0.212 & 0.182 & 0.183 & 0.304 & 0.525 & 0.432 & 0.291 & 0.612 & \mathrm{nan} & 0.292 & 0.366 & 0.456 & 0.785\\ 0.200 & 0.514 & 0.592 & 0.046 & 0.608 & 0.171 & \mathrm{nan} & \mathrm{nan} & 0.966 & 0.808 & \mathrm{nan} & \mathrm{nan} & 0.684\\ \mathrm{nan} & \mathrm{nan} & 0.495 & 0.034 & 0.909 & 0.259 & 0.663 & 0.312 & 0.520 & 0.547 & 0.185 & 0.970 & 0.775\\ 0.939 & \mathrm{nan} & 0.598 & 0.922 & 0.088 & 0.196 & 0.045 & 0.325 & 0.389 & 0.271 & 0.829 & 0.357 & 0.281\\ \mathrm{nan} & 0.141 & 0.802 & \mathrm{nan} & 0.987 & \mathrm{nan} & 0.199 & 0.006 & 0.815 & 0.707 & \mathrm{nan} & 0.771 & 0.074\\ 0.358 & 0.116 & 0.863 & 0.623 & 0.331 & 0.064 & 0.311 & \mathrm{nan} & 0.730 & 0.638 & 0.887 & 0.472 & \mathrm{nan}\\ 0.713 & 0.761 & \mathrm{nan} & 0.771 & 0.494 & 0.523 & 0.428 & 0.025 & 0.108 & 0.031 & 0.636 & 0.314 & 0.509\\ 0.908 & 0.249 & 0.410 & 0.756 & 0.229 & 0.077 & 0.290 & 0.161 & 0.930 & 0.808 & \mathrm{nan} & 0.871 & \mathrm{nan}\\ 0.187 & 0.893 & 0.539 & 0.807 & \mathrm{nan} & \mathrm{nan} & 0.110 & 0.228 & \mathrm{nan} & 0.818 & 0.861 & 0.007 & 0.511\\ \mathrm{nan} & \mathrm{nan} & 0.120 & 0.338 & 0.943 & 0.323 & 0.519 & 0.703 & 0.364 & 0.972 & 0.962 & 0.252 & \mathrm{nan}\\ \mathrm{nan} & 0.285 & 0.037 & 0.610 & 0.503 & \mathrm{nan} & \mathrm{nan} & 0.908 & 0.240 & 0.145 & 0.489 & 0.986 & 0.242\\ 0.672 & 0.762 & 0.238 & 0.728 & 0.368 & 0.632 & 0.634 & \mathrm{nan} & \mathrm{nan} & \mathrm{nan} & \mathrm{nan} & 0.187 & 0.041 \end{array}\right)$

**The matrix after filling**
$\left(\begin{array}{ccccccccccccc} 0.375 & 0.951 & 0.556 & 0.599 & 0.156 & 0.156 & 0.550 & 0.866 & 0.601 & 0.708 & 0.637 & 0.970 & 0.894\\ 0.212 & 0.182 & 0.183 & 0.304 & 0.525 & 0.432 & 0.291 & 0.612 & 0.483 & 0.292 & 0.366 & 0.456 & 0.785\\ 0.200 & 0.514 & 0.592 & 0.046 & 0.608 & 0.171 & 0.399 & 0.749 & 0.966 & 0.808 & 0.488 & 0.652 & 0.684\\ 0.317 & 0.562 & 0.495 & 0.034 & 0.909 & 0.259 & 0.663 & 0.312 & 0.520 & 0.547 & 0.185 & 0.970 & 0.775\\ 0.939 & 0.293 & 0.598 & 0.922 & 0.088 & 0.196 & 0.045 & 0.325 & 0.389 & 0.271 & 0.829 & 0.357 & 0.281\\ 0.267 & 0.141 & 0.802 & 0.329 & 0.987 & 0.379 & 0.199 & 0.006 & 0.815 & 0.707 & 0.508 & 0.771 & 0.074\\ 0.358 & 0.116 & 0.863 & 0.623 & 0.331 & 0.064 & 0.311 & 0.757 & 0.730 & 0.638 & 0.887 & 0.472 & 0.827\\ 0.713 & 0.761 & 0.179 & 0.771 & 0.494 & 0.523 & 0.428 & 0.025 & 0.108 & 0.031 & 0.636 & 0.314 & 0.509\\ 0.908 & 0.249 & 0.410 & 0.756 & 0.229 & 0.077 & 0.290 & 0.161 & 0.930 & 0.808 & 0.341 & 0.871 & 0.712\\ 0.187 & 0.893 & 0.539 & 0.807 & 0.459 & 0.233 & 0.110 & 0.228 & 0.421 & 0.818 & 0.861 & 0.007 & 0.511\\ 0.226 & 0.447 & 0.120 & 0.338 & 0.943 & 0.323 & 0.519 & 0.703 & 0.364 & 0.972 & 0.962 & 0.252 & 0.798\\ 0.123 & 0.285 & 0.037 & 0.610 & 0.503 & 0.189 & 0.216 & 0.908 & 0.240 & 0.145 & 0.489 & 0.986 & 0.242\\ 0.672 & 0.762 & 0.238 & 0.728 & 0.368 & 0.632 & 0.634 & 0.388 & 0.224 & 0.227 & 0.169 & 0.187 & 0.041 \end{array}\right)$

## Appendix C

**The subgroup evaluation matrices**	
$\left[\begin{array}{ccccc} \left(0.06,0.10\right) & \left(0.04,0.12\right) & \left(0.07,0.09\right) & \left(0.09,0.07\right) & \left(0.00,0.15\right)\\ \left(0.12,0.03\right) & \left(0.12,0.04\right) & \left(0.06,0.09\right) & \left(0.06,0.10\right) & \left(0.07,0.09\right)\\ \left(0.15,0.01\right) & \left(0.08,0.08\right) & \left(0.04,0.12\right) & \left(0.06,0.10\right) & \left(0.09,0.07\right)\\ \left(0.09,0.07\right) & \left(0.01,0.15\right) & \left(0.09,0.07\right) & \left(0.06,0.09\right) & \left(0.12,0.04\right)\\ \left(0.06,0.10\right) & \left(0.07,0.09\right) & \left(0.05,0.11\right) & \left(0.11,0.05\right) & \left(0.02,0.14\right) \end{array}\right]$	$\left[\begin{array}{ccccc} \left(0.13,0.14\right) & \left(0.15,0.12\right) & \left(0.20,0.07\right) & \left(0.15,0.12\right) & \left(0.10,0.17\right)\\ \left(0.03,0.24\right) & \left(0.16,0.11\right) & \left(0.20,0.07\right) & \left(0.05,0.22\right) & \left(0.19,0.08\right)\\ \left(0.10,0.17\right) & \left(0.12,0.15\right) & \left(0.07,0.19\right) & \left(0.05,0.22\right) & \left(0.23,0.04\right)\\ \left(0.16,0.11\right) & \left(0.20,0.06\right) & \left(0.13,0.14\right) & \left(0.12,0.15\right) & \left(0.07,0.20\right)\\ \left(0.17,0.10\right) & \left(0.10,0.17\right) & \left(0.15,0.12\right) & \left(0.14,0.13\right) & \left(0.11,0.16\right) \end{array}\right]$
$\left[\begin{array}{ccccc} \left(0.25,0.06\right) & \left(0.18,0.14\right) & \left(0.19,0.12\right) & \left(0.04,0.28\right) & \left(0.16,0.15\right)\\ \left(0.24,0.07\right) & \left(0.19,0.12\right) & \left(0.22,0.09\right) & \left(0.07,0.24\right) & \left(0.14,0.17\right)\\ \left(0.17,0.15\right) & \left(0.18,0.13\right) & \left(0.17,0.14\right) & \left(0.07,0.24\right) & \left(0.17,0.14\right)\\ \left(0.14,0.17\right) & \left(0.20,0.12\right) & \left(0.17,0.14\right) & \left(0.00,0.31\right) & \left(0.11,0.20\right)\\ \left(0.24,0.08\right) & \left(0.22,0.09\right) & \left(0.13,0.18\right) & \left(0.05,0.26\right) & \left(0.13,0.18\right) \end{array}\right]$	$\left[\begin{array}{ccccc} \left(0.11,0.15\right) & \left(0.08,0.18\right) & \left(0.04,0.23\right) & \left(0.16,0.11\right) & \left(0.17,0.09\right)\\ \left(0.19,0.07\right) & \left(0.07,0.19\right) & \left(0.07,0.20\right) & \left(0.16,0.10\right) & \left(0.11,0.15\right)\\ \left(0.19,0.07\right) & \left(0.13,0.13\right) & \left(0.09,0.18\right) & \left(0.20,0.06\right) & \left(0.20,0.06\right)\\ \left(0.18,0.08\right) & \left(0.18,0.08\right) & \left(0.06,0.20\right) & \left(0.11,0.15\right) & \left(0.11,0.16\right)\\ \left(0.18,0.08\right) & \left(0.09,0.17\right) & \left(0.10,0.16\right) & \left(0.13,0.13\right) & \left(0.15,0.11\right) \end{array}\right]$
